# Evolving Partnerships in Community

**DOI:** 10.1289/ehp.7911

**Published:** 2005-07-18

**Authors:** Shobha Srinivasan, Gwen W. Collman

**Affiliations:** Division of Extramural Research and Training, National Institute of Environmental Health Sciences, National Institutes of Health, Department of Health and Human Services, Research Triangle Park, North Carolina, USA

**Keywords:** community-based participatory research, community–university partnership, environmental exposures, health disparities, methodology

## Abstract

In recent years there have been a significant number of publications on the benefits and challenges of community-based participatory research (CBPR). In this introduction we give an overview of three projects presented in this mini-monograph and highlight their commonalities and differences in developing community–university partnerships. While the studies presented here were not required to use CBPR strategies in their work, they did engage community members in a participatory manner. In this mini-monograph we examine how these multifaceted research questions are addressed while simultaneously negotiating complex relationships among researchers and communities as they strive for a more equitable partnership—not only in the distribution of resources but also in power/authority, the process of research, and its outcome. The three papers in this mini-monograph offer insights into various ways of forming, working, and sustaining community–university partnerships in conducting CBPR. They illustrate both the potential benefits and some of the challenges involved with establishing partnerships between community groups and researchers committed to the mutual goal of promoting environmental health. They suggest the importance of nonprescriptive frameworks for conducting community-based participatory research that focuses on more equitable power relationships to address health disparities to help alleviate environmental health problems.

In recent years there have been a significant number of publications on benefits and challenges of community-based participatory research (CBPR) (Shepard et al. 2002). The National Institute of Environmental Health Sciences (NIEHS), through several extramural activities, promotes CBPR that encourages partnerships between community members and academic researchers in public health. Information can be found on the NIEHS web-site (http://www.niehs.nih.gov/translat/home.htm). One of these initiatives is the Health Disparities: Linking Biological and Behavioral Mechanisms with Social and Physical Environments Program (henceforth referred to as the Health Disparities Program, HDP) that was initiated in 2000. The purpose of this program is to foster multidisciplinary research to elucidate underlying mechanisms by which interactions of physical exposure with the social environment lead to health disparities. The research projects in the HDP were based in the community, and requests for application to obtain grants sponsored by the program required partnerships between social/behavioral scientists and biomedical scientists. While the proposed research projects were required to have a community outreach and education program (COEP), they were not required to use CBPR strategies in their work. However, several of the projects funded, including the three highlighted in this mini-monograph series, did engage community members in a participatory manner.

The three articles in this mini-monograph (based in and referred to as the Detroit, Akwesasne, and North Carolina projects) are funded through the HDP. The authors of these articles discuss the partnerships established between communities, community-based organizations, and researchers; the evolution and development of those partnerships in relation to the research effort; the partnerships’ contributions to the evolution and development of the research; and lessons learned in that process. The projects funded within the HDP address complex scientific research questions that try to determine the mechanisms through which the social and physical environments influence biological processes and behaviors that ultimately contribute to health disparities.

The starting point for these research projects is the recognition in the public health community that most human diseases are related in some manner to social factors and forces (Kaplan 1999; Link and Phelan 1995; Schulz et al. 2000; Williams and House 1991). However, the terminology used to describe this relationship is as numerous as the research specialties that study this area (behavioral, social, epidemiologic, etc.).

Diseases may be said to be socially mediated, or to be distributed, patterned, or determined by social factors, as implied in the paragraph immediately above. The presence or distribution of virtually all disease can be related to social factors either in regard to their origin (human impacts on ecology), transmission, or distribution within, between, or among societies. This awareness of the social determinants or dimension of diseases may be the result of a new and better understanding of the etiology of diseases. Understanding disease in contemporary human populations requires in-depth analysis and understanding of the role of social factors. The most complete picture may be obtained by understanding the relevant social forces as much as possible, and linking this knowledge to the biology of disease processes. Because social factors or forces can vary tremendously among and within cultures and societies, obtaining detailed and authentic information of particular social forces may be facilitated through partnerships with community members. Such research requires the involvement of communities at multiple levels—not only to obtain better understanding of the concerns and issues of the communities but also to promote change by involving members of communities who live in these areas and are affected by these processes.

For this type of research, the “community” may or may not be defined geographically, for example, as a neighborhood or town. Because health disparities are prevalent among people of the same socioeconomic class and spread across different municipalities or geographic areas, the community boundary may not coincide with the usual denominators in epidemiologic work. In some cases the community may define itself by using standards of social identification foreign to the researchers or by identifying social boundaries that have not been recognized by the scientific partners but that may be instrumental in disease causation.

The three projects featured in this mini-monograph actively engage members of communities that experience disproportionate burdens of disease or ill health in the following manner.

The Detroit project focuses on race-based residential segregation and its potential link to cardiovascular disease in inner-city Detroit, Michigan. The project also examines the influence of past economic divestment in shaping both physical and social environments that have left the African American and Hispanic communities in substandard housing and in high-crime communities that lack infrastructure.

The Akwesasne project is based in upstate New York, although it includes a significant population that lives in Canada. This project focuses on the effects of polychlorinated biphenyls (PCB) in the St. Lawrence River and how the Mohawk tribe’s culture of interaction with the physical environment exposes the community to pollutants that may affect physical, cognitive, and social well-being. The Akwesasne Mohawk people are faced with a dominant and dominating American culture within which they seek to retain and strengthen their own cultural and religious practices.

The North Carolina project focuses on occupational roots of health disparity among women employed in poultry processing in the rural northeastern region of the state, an area that suffers from a declining local economy. The project examines the interaction of physical exposures at work with the social environment in the workplace. Occupational health and safety issues of African American women, in general, have been poorly researched. More specifically, women employed in the poultry processing industry in the rural South have not been systematically studied.

While all three projects conceptually address physical or chemical exposures, they each emphasize social processes that may influence these exposures—to a greater extent than issues of personal or individual choice. This interaction between physical–chemical exposures and social forces can be much more complicated than mere socioeconomic status. Understanding and appreciation of context are important; for example, the rural women employed in the North Carolina poultry processing industry were not involved in municipal decisions that placed a low-wage company as a dominant employer in their community. Similarly, the Akwesasne people were not in control of the decisions that located toxic waste sites near their tribal lands.

In this mini-monograph we examine how these multifaceted research questions are addressed while simultaneously negotiating complex relationships among researchers and communities as they strive for a more equitable partnership—not only in the distribution of resources but also in power and authority, the process of research, and its outcome. The three projects differ in their study approach; some provide more detail regarding the role of community partners in the development of study design, while others focus on the collaborative process itself. These studies reflect the entire HDP, where some projects had existing structured partnerships with community-based groups while others had identified communities where their research would be based. Some of these projects had more extensive community involvement from conceptualization of the proposal through the outreach component at the end of the project, while others had limited community involvement.

## History of Partnerships

Both the Detroit and Akwesasne projects have longer histories of community–university partnerships with formalized steering committees, established processes for reviewing potential research in the community, publication, and dissemination. The Detroit project can trace its beginnings to the establishment of the Detroit Urban Research Center (URC). In 1995 the Centers for Disease Control and Prevention funded the URC to improve health in selected areas of Detroit through CBPR. The URC board, comprised of representatives from community-based organizations, health service providers, and academic researchers, identified environmental health and social determinants of health as priorities. The Healthy Environments Partnership (HEP) was developed in response to those priorities. Representatives from community-based organizations and local health professionals provided input into the research questions and study design, and several of these people continue as members of the HEP Steering Committee along with some new members. The current Akwesasne project grew from relationships started with the Akwesasne community in the 1980s, whereas the project in the HDP started in 1995. The Akwesasne Mohawk Nation, as a Native American community, has been the subject of numerous studies, and the protracted nature of the relationship with the preexisting partnership was helpful to the current research project. Furthermore, because Akwesasne is a sovereign territory, collaboration developed very deliberately and through a dual process that was familiar on one level to academics and on another level to the Native American community. In the North Carolina project the most recent of the collaborations, the academic researchers were approached by women in the community to address specific issues of concern. Initially, the academic team viewed its role more as one of providing technical assistance to the community. However, as the work and the academic-community partnership evolved, many aspects of CBPR became evident.

## Shared Premises and Evolving Partnerships

Despite marked diversity across various issues, including research topics and methodologies, communities of study and history of collaborations, and diversity of the academic teams, these projects demonstrate a number of shared premises, commitments, and processes. In all three projects there is a shared commitment to scientific rigor to provide credible information, with the recognition that credibility is essential to the ability to negotiate change.

All these research projects required community involvement at various levels and in multiple dimensions. All had community involvement in defining research questions, development of tools, recruitment of participants, and collection of data. In each case the partnership with community members is viewed as essential in obtaining detailed and authentic information about the complex constructs of interest based on the specific aims in each project.

Although the partnerships in all three projects have quite different histories and longevities, these collaborations between the community and academic partners are viewed as a process evolving and, it is hoped, improving over time. This process is analogous to an evolving scientific undertaking in which investigators learn from the work of others and strive to build on their own previous work as well. Because the partnerships between communities and universities are of varying lengths, the partners did not come to the table to discuss the research project with the same levels of experience. Significant time was spent on building relationships based on mutual respect, on establishing the process of communication, and on developing skill (on both the academic and community sides). For example, because in all these projects the primary funding was given to the university partner, significant time was invested in establishing processes by which community members can voice their opinions to influence the research process. Developing a working relationship is a shared and an evolving process because all the project partners need to come to the table to determine how the research will be conducted, develop the protocol, devise data collection methods and procedures, and establish strategies for dissemination and publication. Thus, every issue in the research process is thoroughly discussed together by the community and the academic partners.

When deciding whether there is an equitable distribution of resources, many times community members may not be comfortable requesting salaries higher than those of most of the other community members with whom they work and live. Therefore, it is important that community members decide the appropriate salary or level of reimbursement. In all three projects, community staff and participants are paid for their time, in salary or incentives, as an indication of respect for their valuable time and effort. A higher salary than the earning capacity of other community members may create income disparity and could subsequently lead to differential power among community members. Besides salary it is also important to acknowledge community partnership contributions to public meetings, conferences, presentations, and publications, and to provide opportunities for co-authorship of papers and presentations as well. In recognition of the crucial contribution of communities to the research, the three projects provide funds for travel of community members to various national meetings.

## Conclusion

Some research scientists who use principles of CBPR may be wary of acknowledging that they are indeed involved in CBPR because, in some cases, there was no structured community–university partnership until they received funding for the project. The three projects highlighted in this mini-monograph offer invaluable insights into the ways of forming partnerships and working with communities. Given very different research questions, unique communities, and varying lengths of association with the community, they illustrate a wide range of strategies and processes for working toward establishing mutually respectful scientific collaboration that benefits the communities and researchers alike.

### Partnership structure.

A question that arises in such partnerships is how relationships between partners should be structured. Typical arrangements include subcontracting with community organizations, hiring community members as university employees, and contracting with consultants; each of these possibilities has its challenges and benefits. The three highlighted projects hired community partners as staff members on the project for different reasons. Sometimes community members hired as staff may not feel they have independent voices in the process; however, the experiences in the projects described here show that this method can be successful. The project manager for the Detroit project is a long-time community resident who is also a health professional. In the Akwesasne project the community partner hired works closely with the research team and along with the project’s Steering Committee, which is made up of community members, health professionals, academic researchers, and representatives from the Mohawk community. The addition of the Steering Committee complements the relationships between members of the research team and the community. The North Carolina project in its first year faced difficult decisions when the community organization with which it had partnered was unable to meet its obligations to the community members and the research project. Ultimately, community members who were part of the originally subcontracted community organization left the organization and were hired as staff members on the project. These staff members have had considerable involvement in all aspects of the work, and they were the reason for the current level of success in recruiting research participants, maintaining followup with women in their communities, and developing a wider community outreach.

### Scientific rigor.

Although the ultimate outcome of the research project may be different for the researcher and the community partner, the success of these projects can be linked directly to the commitment of both to scientific rigor and promoting health. Although the community may be more interested in solving and alleviating the present health problems than in the production of scientific knowledge *per se*, their commitment to the research is the result of a focus on addressing immediate health problems. This synergy has resulted in the Akwasasne people developing several research studies with other academic partners. This community is also concerned that the research on the possible human health effects of toxicant exposure be credible within the scientific community because negotiations with industry regarding remediation are still under way. While the ultimate motive for the research may not be identical for communities and researchers alike, scientific rigor is valued by both partners to the extent that it moves forward changes to improve community health and that credibility is essential to negotiate change.

### Power differences.

It is important to note that researchers and communities bring with them, among other issues, unequal power relations and cultural, racial/ethnic, linguistic, and socioeconomic differences, all of which can affect who has influence within the community, as well as who has influence in relation to researchers. While overcoming these differences can be difficult, the challenges can be surmounted through the process of establishing a partnership based on mutual respect. All three projects surmounted these barriers and challenges because it was the community that chose the topic and approached the researchers. In the North Carolina project, the process of allowing members of the existing community organization to be hired as employees on the project group was empowering to those community members because they felt that they consequently had a greater representation and voice in the process of research.

### Sustainability.

One of the biggest concerns for both communities and researchers is sustaining the partnerships beyond the funding period of the grant–especially if there is no financial support for the work. Sustainability of partnerships is extremely challenging and depends ultimately on the abilities of the researchers and the communities to sustain the research or any future endeavors with the relationships they have developed in working together on the basis of shared ideologies.

The three projects described in this mini-monograph offer invaluable insight into forming partnerships and working with community partners, given that they pose very different research questions in different types of communities, and the length of association with the community partners varies widely. These projects illustrate both the potential benefits and some of the challenges involved with establishing partnerships between community groups and researchers committed to the mutual goal of promoting environmental health. Finally, the projects suggest the importance of nonprescriptive frameworks for conducting CBPR that focuses on more equitable power relationships to address health disparities to help alleviate environmental health problems.
